# Immediate Postoperative Analgesia of Nalbuphine-Ketamine Combination Compared with Ketamine Alone in Xylazine-Sedated Goats Undergoing Left Flank Laparotomy

**DOI:** 10.3390/ani12040509

**Published:** 2022-02-18

**Authors:** Mahmoud M. Abouelfetouh, Eman Salah, Lingling Liu, Ahmed H. Khalil, Qiulin Zhang, Mingxing Ding, Yi Ding

**Affiliations:** 1 College of Veterinary Medicine, Huazhong Agricultural University, No.1, Shizishan Street, Hongshan District, Wuhan 430070, China; mahmoud.abouelfetouh@fvtm.bu.edu.eg (M.M.A.); eman.salah@fvtm.bu.edu.eg (E.S.); liulingling678@163.com (L.L.); colynqiulin@163.com (Q.Z.); dmx@mail.hzau.edu.cn (M.D.); 2 Department of Surgery, Radiology and Anaesthesiology, Faculty of Veterinary Medicine, Benha University, Moshtohor 13736, Egypt; ahmed.khalil@fvtm.bu.edu.eg; 3 Department of Pharmacology, Faculty of Veterinary Medicine, Benha University, Moshtohor 13736, Egypt; 4 Clinical Veterinary Laboratory, College of Veterinary Medicine, Henan University of Animal Husbandry and Economy, Zhengzhou 450046, China

**Keywords:** goats, ketamine, nalbuphine, immediate postoperative pain, xylazine

## Abstract

**Simple Summary:**

Goats have been used as animal models in many studies, and therefore, the need for safer anesthesia for research or surgical intervention is gaining much attention. In this current study, we evaluated the quality of anesthesia and the immediate postoperative analgesia of a newer anesthetic combination nalbuphine–ketamine, compared with ketamine alone in xylazine-sedated goats. This combination has been shown to allow the calm, acceptable induction of anesthesia and recovery. In addition, goats anesthetized with nalbuphine–ketamine exhibited a significant decrease in total pain scores postoperatively compared with ketamine. This study concluded that adding nalbuphine to ketamine improves the quality of anesthesia and reduces immediate postoperative pain in goats.

**Abstract:**

Goats have been used as animal models in research, and the need for achieving safer anesthesia for research or surgical intervention is gaining much attention. The objective of this study was to evaluate intraoperative effects and the immediate postoperative analgesia of nalbuphine–ketamine regimen in goats. Twenty clinically healthy adult female crossbred goats weighing 14 ± 2 kg were allocated randomly into each of two equally sized groups. All animals were sedated with intramuscular (IM) xylazine (0.07 mg/kg), then anesthesia was intravenously (IV) induced with ketamine alone (10 mg/kg) (XK group), or a combination of nalbuphine (0.5 mg/kg) and ketamine (5 mg/kg) (XNK group). Following induction, left flank laparotomy was performed and then sutured. The quality of anesthesia and immediate postoperative analgesia was evaluated. Immediate postoperative analgesia was assessed up to 5 h after standing, using a modified Unesp–Botucatu acute composite pain scale (USAPS). Serum cortisol, glucose, insulin, and C-reactive protein (CRP) were measured at ½, 1, 2, 4, 6, 12, and 24 h, postoperatively (PO). The USAPS pain scores were significantly lower in the XNK compared to the XK group (*p* < 0.05). The XNK group exhibited a statistically significant difference in the level of serum cortisol at ½ and 1 h PO (*p* = 0.018 and 0.045, respectively) compared to the XK group. At 2, 4, 6 h PO, CRP significantly decreased (*p* = 0.023, 0.040 and 0.005, respectively) in the XNK compared to the XK group. Nalbuphine–ketamine produced an acceptable induction of anesthesia and recovery compared to ketamine. Recovery with nalbuphine–ketamine was faster and better quality. The USAPS pain scores were lower in nalbuphine–ketamine, indicating that this novel combination produces better postoperative pain control than ketamine alone.

## 1. Introduction

Goats are progressively being used in research as animal models [1], so the need for anesthesia with effective pain control for the surgical treatment or research of these animals is gaining much interest. The current progress in comprehending the neurophysiologic basis of pain, with respect to transduction, transmission, modulation, and perception, and the emergence of a wide variety of analgesics, has guided researchers and clinicians to propose that multimodal analgesia can successfully control pain. Multimodal analgesia refers to the concept of combining several analgesic agents to control pain via acting at different targets along the nociceptive system. The importance of this strategy lies in its synergistic analgesic property that enhances intra-and postoperative analgesia and improves patient well-being. Additionally, smaller doses of each agent are usually required, thereby optimizing hemodynamics and reducing the likelihood of the development of its side effects [2,3,4].

Nalbuphine, 17-(cyclobutylmethyl)-4,5-α-epoximorphinan-3,6α,14-triol, is a partial opioid agonist which is structurally similar to oxymorphone. It is an antagonist at μ opioid receptors (OR), while achieving analgesia via an agonistic activity on κ OR. Nalbuphine is proven to induce comparable analgesia as morphine when given in equal doses [5]. In humans, nalbuphine is regarded as an important element of multimodal anesthesia and used as a pain-relieving medication for moderate and severe conditions, preoperative and postoperative analgesia, and gynecological interferences [6]. Nalbuphine has not been extensively used in veterinary practice, however, some studies of the use of nalbuphine have been reported in cats [7], dogs [8,9], horses [10], and camels [11], disclosing that nalbuphine could produce a superior analgesia without affecting cardiopulmonary variables.

Ketamine is a dissociative anesthetic drug that has remained the principal component in anesthesia management for small ruminants, due to its affordable cost, analgesia, and wide safety margin [12], however it is associated with excitatory signs during recovery. Therefore, it is usually co-administered with other adjuncts, such as benzodiazepines [13], opioids [14,15], and α_2_-adrenergic agonists [16], in an effort to improve muscle relaxation and reduce the required dose of ketamine. Xylazine is an α_2_ adrenergic agonist that is most commonly used in veterinary practice for its sedative, analgesic, and muscle relaxant properties. Its actions were mediated via α_2_ adrenergic receptors distributed centrally in the brain or supraspinally (for sedation and some analgesia), and in the dorsal horn of the spinal cord (for analgesia) [17]. In ruminants, a lower dose of xylazine is needed to induce same analgesic and sedative effects as in other domestic species, such as horses, donkeys, and dogs [18]. Additionally, a dose-dependent depression in cardiopulmonary function could be associated with xylazine administration in ruminants [19].

Alpha_2_ adrenergic agonists, opioids, and ketamine could act synergically to produce multimodal analgesia. Therefore, the objective of this report was to assess intraoperative and immediate postoperative analgesia, along with the quality of anesthesia induced with IV nalbuphine–ketamine combination, compared with ketamine alone in xylazine-sedated goats undergoing left flank laparotomy. Our assumption was that nalbuphine and ketamine regimen at a dose of 0.5 mg/kg and 5 mg/kg, respectively, would enhance the anesthetic quality, and improve the immediate postoperative analgesia compared to ketamine alone, at a dose of 10 mg/kg in goats.

## 2. Material and Methods

### 2.1. Animals

Twenty clinically healthy adult female crossbred goats ranging from 6–8 months old, and weighing 14 ± 2 kg, were involved in this study. The animals were purchased locally, and their physical status was ensured through a comprehensive medical examination, including cardiothoracic auscultation, ECG, and testing for packed cell volume (PCV), complete blood count (CBC), and serum biochemical profile. Goats were brought into an experimental research unit and kept under a suitable environmental condition. Goats were excluded if they showed evidence of systemic diseases and/or aggressiveness on clinical examination. The animals were acclimatized to handling and the environmental condition one week prior to the experiment. Food, but not water, was withheld overnight before starting the experiment. This study was approved by the animal experimental ethical inspection of the Laboratory Animal Center, College of Veterinary Medicine, Huazhong Agricultural University (ID number: HAZUGO-2021-0002).

### 2.2. Experimental Design

Goats were randomly assigned to one of two equally sized groups using a computer program (www.randomizer.org, accessed on 6 December 2021). In this randomized, blind, and experimental trial, xylazine was administered for sedation, and either ketamine (XK group) or nalbuphine–ketamine (XNK group) was administered for the induction of anesthesia. In the XK group, goats were sedated with intramuscular (IM) xylazine (Xylaject 2%, Adwia, Egypt) at a dose of 0.07 mg/kg. Ten min after xylazine administration, anesthesia was induced with ketamine alone (10 mg/kg) or a combination of nalbuphine (0.5 mg/kg) and ketamine (5 mg/kg), as an intravenous (IV) bolus over 10 s through the jugular vein.

Prior to induction, a 20-gauge 2.5-cm catheter was placed in each goat’s left jugular vein, and the left flank regions of all goats were shaved and aseptically prepared for surgery. Goats were allowed to breathe room air before, during, and after induction, and placed in the right lateral recumbency immediately after induction. Two min after induction, left flank laparotomy was done, and then closed using a standard surgical procedure [20]. A seven centimeter (7 cm) incision was made over the left flank region. Then, the incision was sutured in two layers using an Ethicon Vicryle of the size of 2–0 in a simple continuous suture pattern: peritoneum and transverse abdominal muscle, internal and external abdominal muscles. The skin was closed using Ford interlocking pattern with 1–0 silk. Heart rate (HR; beats/min), respiratory rate (*f*_R_, breaths/min), hemoglobin oxygen saturation (SpO_2_, %), and rectal temperature (RT, Co) were recorded using a multiparameter ECG monitor (PM-9000 Express, Mindary Co., Ltd., Shenzhen, China) before sedation (baseline), during surgery (TS), at the end of surgery [(TE (0)], and at 10 min intervals, until the goat voluntarily moved. Once the goat moved, the ECG was removed, except for the jugular catheter, and the goats were allowed to recover unassisted. The baseline values were recorded while the goat was in a standing position.

### 2.3. Induction and Recovery Assessment

Induction and recovery characteristics were judged by an experienced anesthetist, who was unaware of the treatment given, using a modified numerical scoring scale of 0–2 (0: good, 1: fair: 2: poor) (Appendix A) [21]. Times to first movement and standing were recorded.

### 2.4. Immediate Postoperative Analgesia Assessment

Blood specimens were collected from the jugular catheter at baseline, TInd, TE (0), and at ½, 1, 2, 4, 6, 12, and 24 h postoperatively for biochemical measurement of cortisol, glucose, insulin, and C-reactive protein (CRP). Blood for cortisol, insulin, and CRP was collected into gel and clot activator tubes and left for 10 min in a slanted position to coagulate before centrifugation at 1200× *g* for 20 min to obtain serum. Blood for glucose was collected in potassium fluoride and Na_2_ EDTA tubes and centrifuged immediately at 1200× *g* for 20 min to obtain plasma. Cortisol, insulin, and CRP were assayed using specific goat analytical Eliza kits purchased from Bioassay Technology Laboratory (BT LAB), Yangpu Dist., Shanghai, China.

After standing subjective pain scores were assessed using a modification of a recently published Unesp–Botucatu sheep acute composite pain scale (USAPS) [22] every ½ up to 5 h by a trained observer blind to the treatments. This method includes the valuation of behavioral indicators of pain (interaction, locomotion, head position, posture, activity, and appetite) assigning a scale of 0–2 for each criterion. Therefore, a score of 10 denotes maximum pain, and a score of zero represents no pain (Appendix B).

### 2.5. Statistical Analysis

A statistical analysis was carried out with Graphpad Prism software version 8.0 (GraphPad Inc, San Diego, CA, USA). Ordinal data (pain scores and induction and recovery scores) and recovery times (first movement and standing) were presented as median and interquartile range (IQR) and continuous data (physiologic parameters and biochemical measures) were reported as mean ± SD. The Kolmogorov–Smirnov test was used to assess the normality (Gaussian distribution) of variables. Two-way repeated measures ANOVA with Bonferroni’s post-hoc test was used to compare variables within and between groups. Wilcoxon matched-pairs signed-rank test was used to compare variables with categorical data (scores) and recovery times between groups. The correlation between cortisol, glucose, and CRP was analyzed using Pearman correlation analysis. A significant difference was supposed when *p* value < 0.05.

## 3. Results

### 3.1. Induction and Recovery Qualities

In this study, induction score was 1 (0–1) in the XNK group and 1 (1–2) in the XK group, and there were significant differences between two groups (*p* = 0.031). Muscle fasciculation and limb paddling, and the extensive backward deviation of the head and neck, were predominantly observed during induction with ketamine, compared with nalbuphine–ketamine. The recovery score was significantly lower in the XNK group compared to the XK group [0.5 (0–1) and 2 (1–2), respectively, *p* = 0.047). In the XNK group, goats recovered calmly from anesthesia and exhibited an easy transition from recumbency to standing position compared to those in the XK group. Time to first movement in the XK group [34.5 (32.3–41.5)] was not different from that of the XNK group [37.0 (35.75–41.25)]. However, goats in the XK groups exhibited a longer time to standing [55.0 (49.5–56.8) compared to those in the XNK group [38.0 (36.8–47.8)] (*p* = 0.002). In this study, nalbuphine–ketamine or ketamine created an anesthetic state convenient for performing left flank laparotomy. Goats showed no movement during the time of surgery in the XK and XNK group [6.27 (6.14–6.41) and 6.32 (6.22–6.42) min, respectively] (Table 1). So, no additional increments of anesthetics are required in either group.

### 3.2. Immediate Postoperative Analgesia Assessment

**Figure 1:**
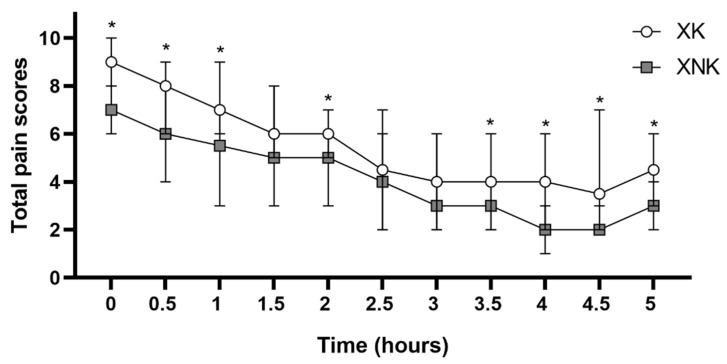
Median and range of total pain scores in the XK group (*n* = 10) and XNK group (*n* = 10) assessed over the 5 h after standing (0) obtained using Unesp–Botucatu composite pain scale (USAPS) modified after Silva et al., 2020. * Significant difference between the two groups at the same time point (*p* < 0.05).

The results of the subjective pain evaluations over the 5 h after standing are shown in Figure 1. Compared to the XK group, the XNK exhibited a lower pain score throughout the postoperative period, with statistically significant decreases observed at the first observation (standing time), 0.5, 1, 1.5, 2, 2.5, 3, 3.5, 4, 4.5, and 5 h after standing.

**Figure 2:**
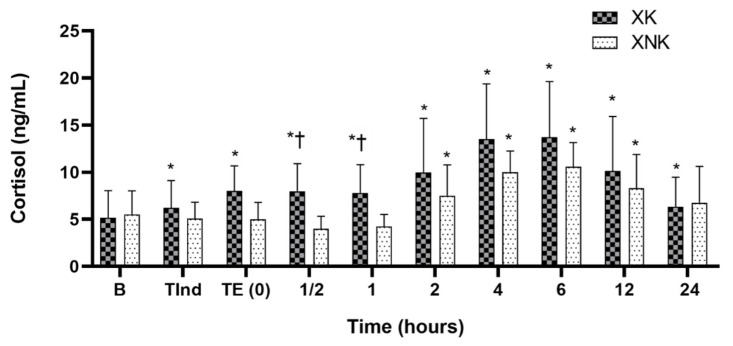
Mean ± SD of serum cortisol concentration (ng/mL) in the XK (*n* = 10) and XNK group (*n* = 10). * Significant difference compared to baseline within each group (*p* < 0.05). † Significant difference between the two groups at the same time point (*p* < 0.05).

Serum cortisol concentration (ng/mL) at TInd, TE (0) and at ½, 1, 2, 4, 6, 12, and 24 h, after the end of left flank laparotomy in both groups is presented in Figure 2. The serum cortisol was significantly higher in the XK group at TInd, TE (0), and at ½ to 6 h after the end of surgery compared to the baseline, then began to decrease, and become significantly lower at 12 and 24 h after the end of surgery. In the XNK group, cortisol showed non-significant differences at TE (0) and at ½ to 1h after the end of surgery, in comparison with the baseline; however, a significant increase was detected at 2, 4, 6, and 12 h after the end of surgery. In both groups, cortisol level was considered to have returned to normal at 24 h postoperatively. Compared to the XK group, the XNK group exhibited a lower level of cortisol at all time points, with a statistically significant difference observed at ½ and 1 h, postoperatively (*p* = 0.018 and 0.045, respectively).

**Figure 3:**
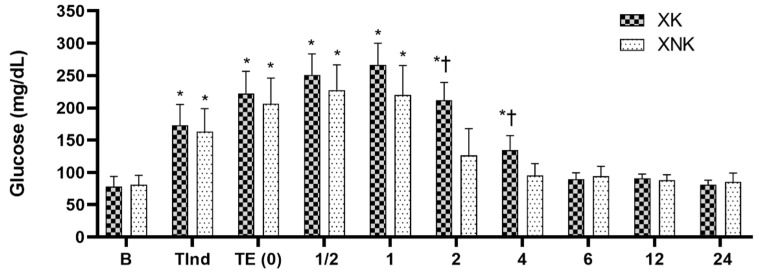
Mean ± SD of plasma glucose (mg/dL) in the XK (*n* = 10) and XNK group (*n* = 10). * Significant difference compared to baseline within each group (*p* < 0.05). † Significant difference between the two groups at the same time point (*p* < 0.05).

The mean values obtained for plasma glucose (mg/dL) in the XK and XNK group are shown in Figure 3. In both groups, glucose showed an increasing trend from anesthetic induction to 1h postoperatively, and afterwards, it decreased gradually and become constant at 6, 12, and 24 h, postoperatively. Compared to baseline, a significant increase was observed at TInd, TE (0), and ½, and 1 h postoperatively in both groups (*p* < 0.001). The XNK group showed a significant decrease in the glucose level at 2 and 4 h postoperatively, compared to the XK group.

**Figure 4:**
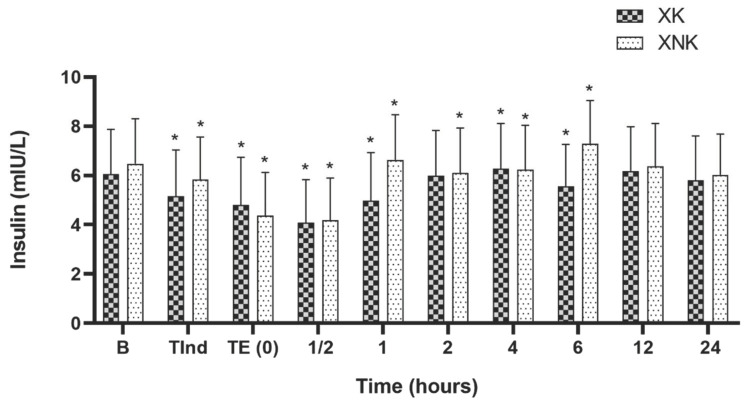
Mean ± SD of serum insulin concentration (MIU/ mL) in the XK (*n* = 10) and XNK group (*n* = 10). * Significant difference compared to baseline within each group (*p* < 0.05).

The serum insulin level (MIU/mL) decreased gradually after induction in both groups to reach the minimum level at ½ h postoperatively (*p* < 0.05). The level began increasing and decreasing inconsistently around the baseline value till 6 h postoperatively and become stable and comparable to the baseline at 12 and 24 h after the end of surgery. Non-significant differences observed in the insulin concentration between groups (Figure 4).

**Figure 5:**
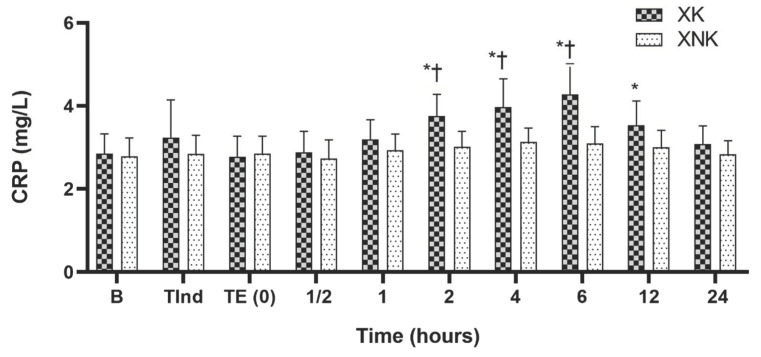
Mean ± SD of serum C-reactive protein (mg/L; CRP) concentration in the XK (*n* = 10) and XNK group (*n* = 10). * Significant difference compared to baseline within each group (*p* < 0.05). † Significant difference between the two groups at the same time point (*p* < 0.05).

No significant changes occurred in the CRP concentration (mg/L), either at TInd or at any time point postoperatively compared to the baseline in the XNK group. However, in the XK group, a significant increase in CRP was found at 2, 4, and 6 h postoperatively (*p* = 0.003, 0.008, and 0.002, respectively). Compared to the XK group, the XNK group exhibited a significant decrease in CRP at 2, 4, and 6 h postoperatively (*p* = 0.023, 0.040, and 0.005, respectively), which positively correlated with the cortisol level (*r* = 0.72) (Figure 5).

### 3.3. Basic Physiological Parameters

There were no differences in HR and *f*_R_ compared to baseline within XK and XNK groups. However, in the XK group, a significant increase in *f*_R_ was observed at 20 min after the end of surgery (*p* = 0.028). SpO_2_ was above 90% in both groups, but a significant decrease was found at TInd, TS, TE (0) and 10, 20, and 30 min after the end of surgery in the XK group (*p* < 0.05) and at TS in the XNK group (*p* = 0.037), compared with baseline. RT lowered greatly (*p* < 0.05) within groups. Compared with the XK group, the XNK showed non-significant differences in HR, SpO_2,_ and RT at all time points, however a significant decrease in *f*_R_ was observed at 20 and 30 min postoperatively (Table 2).

## 4. Discussion

Multimodal analgesia, referred to using multiple analgesic agents, has been widely accepted in veterinary medicine to synergically control nociception intraoperatively, and pain postoperatively [23,24]. In this current study, the anesthetic quality, intraoperative effect, and the immediate postoperative analgesia of a novel intravenous (IV) induction protocol of nalbuphine (0.5 mg/kg) and ketamine (5 mg/kg) combination, were evaluated in comparison with ketamine alone (10 mg/kg) in xylazine-sedated goats undergoing left flank laparotomy. Opioid agonist–antagonist analgesics as nalbuphine and butorphanol have been demonstrated to provide potent analgesia, as well as possessing minimal side effects. Therefore, it is beneficial to incorporate these drugs as part of multimodal pain therapy. Nalbuphine is considered equipotent with morphine when administered parenterally [25]. Therefore, the dose nalbuphine used in the present study was chosen based on the analgesic dose of morphine reported in dogs (0.5 mg/kg) [26].

The induction and recovery quality scores were significantly lower in goats that received nalbuphine–ketamine compared to ketamine, and this finding is in agreement with studies in camels and calves [11,27]. In this current study, goats administered with nalbuphine–ketamine showed mild ataxia, uncomplicated transition to alertness, and minimal coordinated attempts to stand than those administered with ketamine alone. The administration of ketamine results in the depression and dissociation of both thalamocortical and limbic systems causing emergence delirium and changes in patient awareness to the surrounding environment [28]. Our findings suggest that nalbuphine–ketamine combination may be preferred over ketamine when rapid and uneventful induction and recovery are important. As well, no ataxia was observed in bucks during recovery from epidurally injected nalbuphine [29]. As well, nalbuphine delivered at high doses during anesthesia has been exhibited to induce a fast recovery in humans [30]. The calm recovery without agitation associated with nalbuphine administration could be attributed to the fact that nalbuphine primarily acts on c-fiber nociceptors, not motor or sympathetic receptors [25]. In cats, nalbuphine-based anesthetic combination has also been shown to provide superior clinician satisfaction during gonadectomy [7].

In this current study, the xylazine-sedated goats received nalbuphine–ketamine showed a lower immediate postoperative pain scores compared to ketamine. In previous studies, nalbuphine has been reported to reduce stress-associated behaviors in calves [27], camels [11], and dogs [8]. Additionally, adding nalbuphine to xylazine appears to enhance analgesia and decrease distress in dogs undergoing an uncomfortable or painful interference [31]. Epidural nalbuphine significantly lowered pain scores on the visual analogue system (VAS) and Colorado pain scales, and provided prolonged postoperative analgesia in dogs [32]. Nalbuphine and butorphanol are both mixed opioid agonist–antagonists with nearly similar pharmacological properties. In this current study, nalbuphine has been shown to produce analgesia, decrease restlessness, and improve behavioral outcome comparable to the effect of butorphanol in goats [21,33,34].

No changes occurred in HR over time in goats that received either nalbuphine–ketamine or ketamine. In prior studies, a decrease in HR was associated with other anesthetic combinations, such as xylazine–ketamine–diazepam, xylazine–propofol and xylazine–thiopentone [35,36]. Although a statistically significant decrease in SpO_2_ compared to baseline was observed more in ketamine than in nalbuphine–ketamine, the level of SpO_2_ remained > 90% in both inductions. This is consistent with that observed after midazolam–butorphanol–alfaxalone induction in goats [21] and medetomidine–alfaxalone in sheep [37]. In this current study, RT were decreased in both nalbuphine–ketamine and ketamine inductions, and non-significant difference detected between the two inductions. The decrease in RT might be explained by xylazine sedation, which induces thermoregulatory centers, as well as reducing the metabolic rate [38].

Serum cortisol is considered an objective reliable indicator of pain and stress during and after surgery. The stress response could stimulate the hypothalamic pituitary adrenal axis, which results in an increase in cortisol release. Cortisol level has been found to significantly increase after surgical stimuli in goats [39], cattle [27], dogs [40], and cats [41]. In this current study, the XNK group displayed a non-significant increase in cortisol level at TInd, TE (0), and ½, 1 h postoperatively, however the XK group significantly increased the cortisol at the same timepoints compared to baseline. Compared to the XK group, the XNK group showed a lower level of cortisol at TInd, TE (0), and ½, 1, 2, 4, 6, 12, and 24 h postoperatively, with a statistically significant decrease detected at ½ and 1 h timepoints. In prior studies, nalbuphine has been reported to enhance postoperative analgesia and decrease surgical stress and pain [7,31,42], which could be pivotal for reducing cortisol release. In this current study, the subjective immediate postoperative pain score almost correlated with the dynamic alteration which occurred in the serum cortisol concentration in both inductions. Nalbuphine and morphine have been reported to possess virtually similar half-life (h), clearance (ml/min/kg), and volume of distribution (l/kg) (1.2 and 1.2; 60 and 46; 4 and 4.6, respectively) [25]. In this current study, the maximum duration of analgesia produced by nalbuphine is from 1–2 h, comparable to morphine given at the same dose in dogs, corresponding with the identical pharmacokinetic profile of both drugs [26,43].

Glucose has been used as a measurement tool for stress and pain in cats [42] and human infants [44]. In this current study, a significant increase in glucose level was observed at TInd, TE (0), and ½ h and 1 h postoperatively, compared to baseline in both groups (*p* < 0.001). Then, the level decreased gradually and become constant at 6, 12, and 24 h, postoperatively. The XNK group showed a significant decrease in the glucose level at 2 and 4 h postoperatively, compared to the XK group. Despite these differences, glucose levels were negatively correlated with cortisol levels, and were not considered useful markers of pain or stress [42,45]. Catecholamines released during stressful procedures lead to increased glucose levels to meet increasing metabolic demands [45]. As well, increasing the glucose level at the following inductions can be attributed to the hyperglycemic effect of ketamine and xylazine [46,47,48]. A significant decrease in insulin level was also reported in both groups. In previous studies, xylazine has been shown to induce hyperglycemia and hypoinsulinemia in cattle [49] and horses [50]. The reported hyperglycemia following the use of xylazine is probably due to a reduction in insulin release from the β-cells in the pancreas and/or an increase in glucagon release from the α-cells [51,52].

CRP is an acute phase protein, which is released in response to surgical trauma and inflammation, and its serum concentration may truly reflect the extent of surgical stress and pain [53,54]. The release of CRP increased proportionally with the degree of trauma and presence of pro-inflammatory cytokines in circulation [55]. In this present study, a significant decrease in CRP was observed at 2,4, 6 h postoperatively in the XNK compared to the XK group. However, in prior reports, CRP has been shown to significantly increase postoperatively in dogs undergoing different surgical interventions [54,56]. The reduction of CRP may be attributed to the decreasing effect of nalbuphine on peripheral cytokines [57].

This current study could provide valuable information about immediate the postoperative analgesia of nalbuphine–ketamine combination compared with ketamine alone in xylazine-sedated goats. Between both inductions, non-significance changes were observed among the measured parameters. Therefore, additional studies are encouraged to further confirm the findings observed in this study.

## 5. Conclusions

Nalbuphine (0.5 mg/kg) and ketamine (5 mg/kg) combination produced acceptable induction anesthesia and recovery compared to ketamine (10 mg/kg) in xylazine-sedated goats undergoing left flank laparotomy. Recovery with nalbuphine–ketamine was faster and of better quality. The immediate postoperative USAPS pain score was lower in nalbuphine–ketamine compared with ketamine. Our findings suggest that nalbuphine–ketamine combination may be preferred over ketamine when enhanced immediate postoperative analgesia as well as uneventful induction and recovery are important.

## Figures and Tables

**Table 1:** Time of surgery, induction and recovery scores, times to first movement and standing in xylazine-sedated goats undergoing flank laparotomy anesthetized with ketamine (10 mg /kg; IV) (XK group), or a combination of nalbuphine and ketamine (0.5 and 5 mg/kg; IV) (XNK group).

Parameter	XK Group (*n* = 10)	XNK Group (*n* = 10)	*p* Value
Time of surgery (min)	6.27 (6.14–6.41)	6.32 (6.22–6.42)	
Induction score	1 (1–2)	1 (0–1) *	0.031
Recovery score	2 (1–2)	0.5 (0–1) *	0.047
Time to first movement (min)	34.5 (32.25–41.5)	37.0 (35.75–41.25)	
Time to standing (min)	55.0 (49.5–56.8)	38.0 (36.8–47.8) *	0.002

Data are expressed as median and interquartile range (IQR). * Significant difference between the two groups (*p* < 0.05).

**Table 2:** Basic physiological parameters in xylazine-sedated goats undergoing flank laparotomy anesthetized with ketamine (10 mg /kg; IV) alone (XK group) or a combination of nalbuphine and ketamine (0.5 and 5 mg/kg; IV) (XNK group). Data were obtained at baseline (before treatment (s), immediately after induction (TInd), during surgery (TS), at the end of surgery [TE (0)] and at 10, 20, and 30 min postoperatively).

Parameter	Group	Time Points (Minutes)						
		B	T Ind	TS	TE (0)	10	20	30
HR (beats/min)	XK	110 ± 13.4	114 ± 8.5	102 ± 12	105 ± 9.8	102 ± 10.4	106 ± 10.1	111 ± 6
	XNK	107 ± 11.3	110 ± 10.8	104 ± 10.2	107 ± 15	107 ± 12.8	105 ± 10.2	100 ± 11.4
*f*_R_ (breaths/min)	XK	21 ± 5	24 ± 4.7	24 ± 4.6	26 ± 5.3	23 ± 5.7	33 ± 11.2 *† † *p* = 0.006	26 ± 4.8 † *p* = 0.001
	XNK	21 ± 4.2	20 ± 3.8	22 ± 3.2	20 ± 3	19 ± 3.3	16 ± 3.6	17 ± 3
SpO_2_ (%)	XK	97 ± 1.7	94 ± 1.6 * p = 0.009	90 ± 4.3 * p = 0.006	91 ± 2.7 * p = 0.001	92 ± 2.6 * p = 0.002	92 ± 2.9 * p = 0.001	91 ± 3.7 * p = 0.014
	XNK	96 ± 2.9	95 ± 2.4	93 ± 2.2 *	93 ± 2.3	93 ± 2.8	94 ± 2	93 ± 1.7
RT (°C)	XK	39.4 ± 0.19	39.3 ± 0.21	39.3 ± 0.26	39 ± 0.18 * p = 0.001	38.9 ± 0.18 * p < 0.001	38.8 ± 0.16 * p < 0.001	38.9 ± 0.13 * p < 0.001
	XNK	39.2 ± 0.19	39.1 ± 0.17 * p = 0.004	39.1 ± 0.14 * p = 0.023	39 ± 0.12	38.9 ± 0.12 * p = 0.001	38.8 ± 0.12 * p = 0.001	38.8 ± 0.15 * p = 0.001

Heart rate (HR), respiratory rate (*f*_R_), hemoglobin oxygen saturation (SpO_2_), and rectal temperature (RT). Data were expressed as mean ± SD. * Significant difference compared to baseline within the group (*p* < 0.05). † Significant difference between the two groups (*p* < 0.05).

## Data Availability

The data set used for statistical analysis is available upon reasonable request.

## Appendix A

**Table A1:** Criteria used for assessment of quality of induction anesthesia with ketamine or nalbuphine–ketamine in xylazine-sedated goats.

Scoring	Description	Score
Induction	Smooth induction, rapid recumbency, no signs of excitement.	0 Good
	Slightly prolonged, mild excitation, some rapid blinking, some limb movements.	1 Fair
	Obvious excitement, muscle twitching; paddling of limbs, extensive backward deviation of head and neck and/or attempts to stand after recumbency.	2 Poor
Recovery		
	Smooth, easy transition to alertness, able to walk with minimal ataxia, minimal attempts to stands (1–2 coordinated attempts).	0 Good
	Transient excitement, or whole-body movements, some struggling, moderate ataxia and several coordinated attempts to stand.	1 Fair
	Stereotypical behavior, circling, prolonged struggling, several premature (uncoordinated) attempts to stand.	2 Poor

Modified from Dzikiti et al., 2014 [21].

## Appendix B

**Table A2:** Unesp–Botucatu sheep acute composite scale (USAPA) for the assessment of immediate postoperative analgesia of ketamine or nalbuphine–ketamine in xylazine-sedated goats.

Criterion	Description	Score
Interaction	Active, attentive to the environment, interacts and/or follows other animals.	0
	Apathetic: may close to other animals but interacts little.	1
	Very apathetic: isolated or not interacting with other animals, not interested in the environment.	2
Locomotion		
	Moves about freely, without altered locomotion; when stopped, the pelvic limbs are parallel to the thoracic limbs.	0
	Moves about with restriction and/or short steps and/or pauses and/or lameness; when stopped, the thoracic or pelvic limbs may be more open and further back than normal.	1
	Difficulty and/or reluctant to get up and/or not moving and or walking abnormality and/or limping; may lean against a surface.	2
Posture		
	Arched back.	
	Extends the head and neck.	
	Lying down with head resting on the ground or close to the ground.	
	Moves the tail quickly (except when breast feeding) and repeatedly and/or keeps the tail straight (except to defecate/urinate).	
	Absence of these behaviors.	0
	Presence one of the related behaviors.	1
	Presence of two or more of the related behaviors.	2
Activity		
	Moves normally.	0
	Restless, moves than normal or lies down and get up frequently.	1
	Moves less frequently or only when stimulated using a stick or does not move.	2
Appetite		
	Normorexia and /or rumination.	0
	Hyporexia.	1
	Anorexia.	2
Total		0–10

Modified after Silva et al., 2020 [22].
